# AI as a peer reviewer: who and how evaluate manuscript value?

**DOI:** 10.1093/ehjimp/qyag118

**Published:** 2026-06-24

**Authors:** Shigeki Matsubara

**Affiliations:** Department of Obstetrics and Gynaecology, Jichi Medical University, 3311-1 Yakushiji, Shimotsuke, Tochigi 329-0498, Japan

**Dear Editors,**

Introduction of artificial intelligence into peer review has prompted us to reconsider what manuscript value is and how it should be evaluated.

Zancanaro *et al*.^1^ showed interesting data. In 40 manuscripts (20 accepted, 20 *de novo* rejected) and 118 peer reviews from a cardiology journal, 67.5% of AI-generated reviews matched the final editorial decision, compared with 71.9% of human reviews. They interpreted this as evidence of non-inferiority of AI peer review. They supported the integration of AI as a ‘complementary tool’ in editorial workflows.

Acceptance or rejection does not always reflect the ‘value’ of a manuscript. Having spent approximately half a century in clinical practice and medical writing, I have published more than 560 first-authored papers. Some were accepted immediately, whereas others were accepted only after submission to a ninth journal. One manuscript was rejected by a Q3 journal and, after only minor formatting changes, it was accepted by a Q1 journal.^2^ Acceptance and rejection often depend on factors other than scientific value, including journal–manuscript matching, journal policy, and timing. For example, when journals receive several manuscripts on a similar topic, some may welcome them as a series whereas others may regard the available space as already occupied. My experience is merely an *N* = 1 observation and thus not persuasive evidence. However, it is time tested. If one does not adhere excessively to impact factor or manuscript type and has the patience to wait for the right opportunity, many manuscripts may eventually find a home somewhere.^2^

Thus, using actual acceptance or rejection as the reference standard does not necessarily evaluate whether AI correctly identifies manuscript value. I understand that there is currently no practical alternative, since the ‘true value’ of a manuscript cannot be determined, especially at the time of submission. However, this raises an interesting question. If in future, more advanced AI systems show substantial disagreement with human reviewers, should that automatically be interpreted as inferiority? AI may identify the value of a manuscript differently, and we cannot deny the possibility that AI could do it even better than humans.

This leads to a more fundamental issue: what is the value of a manuscript? The answer may vary among individuals. Tentatively, in medical publishing, value may be considered a combination of novelty and usefulness to patients. If an experienced editor instructed AI to evaluate a manuscript explicitly from these perspectives, AI might produce conclusions that differ from conventional human review. In such a situation, which should carry greater weight: the conventional but individualized human assessment or the AI assessment?

If AI can recognize hidden value better than humans, should we give greater consideration to its judgement? Yet who should determine whether the value identified by AI is truly valuable, and by what criteria? The matter becomes even more complicated because the definition of value itself may change over time.

Error is human. Even experienced reviewers and editors may misjudge manuscripts. We have no definitive criteria for acceptance or rejection, and we do not even have a universally accepted definition of value. In the AI era, that definition may continue to evolve.

I, slightly changing the topic, wish to touch my small proposal to ‘tentatively’ ameliorate the current problem. The shortage of reviewers is a real problem, which is one of the main reasons for Zancanaro *et al*.’s study. Having served as editor-in-chief of several journals, I understand the difficulties journals face. I was taught that if one publishes 10 papers, one should perform at least 20 reviews. The rationale is simple: even if all 10 papers are accepted on first submission, each required the voluntary efforts of at least two reviewers. Publishing should be therefore a give-and-take process. I have tried to follow this advice and have reviewed more than 2800 manuscripts. Encouraging, or even requiring, publishing authors to accept review invitations may provide a tentative measure to reduce the burden on journals.

Let us return to the present context. Research on AI peer review should continue. Zancanaro *et al*. provided important factual data. This study prompted us to consider human–AI ‘cooperation’ scenario in peer review: AI viewpoint and human viewpoint might be, or will become, different. Then, AI review, in its real meaning, becomes a peer review tool ‘complementary’ to humans. AI may serve as a lens through which we reconsider a more fundamental question: what is a valuable paper?

## Data Availability

Data sharing is not applicable to this article as no new data were created or analysed in this study.

## Lead author biography

Shigeki Matsubara served as the Director of the Department of Obstetrics and Gynaecology at Jichi Medical University (Tochigi, Japan) and Senior Vice President of Jichi Medical University Hospital. After retirement, he has continued his academic work as Emeritus and Visiting Professor.

His major fields include obstetric care, obstetric cardiology, rural medicine, medical writing, and medical artificial intelligence. He has authored more than 560 PubMed-indexed papers as first author and has written two books on medical writing.
